# The Sequelæ of Enteric Fever—II

**Published:** 1908-06-13

**Authors:** 


### Medicine.

### THE SEQUELÆ OF ENTERIC FEVER—II.

Having discussed slow convalescence and temporary œdema of the ankles, which are the only common sequelæ of typhoid fever, it remains to discuss others which, though not likely to occur in any given case, may yet be encountered sooner or later.

### Post-Typhoidal Suppuration.

First and foremost amongst these, and particularly important because of the perpetual possibility of the dissemination of the disease by reason of them, come post-typhoidal suppurations. The best known of these is probably empyema of the gall-bladder; but others that should be borne in mind are pyuria, otitis media and otorrhœa, cutaneous boils, submammary abscess, and suppuration connected with the long bones.

One of the very remarkable facts about the typhoid bacillus is its apparent power, when it gives rise to pus, of preventing the locality already infected by it from being attacked, or at any rate successfully invaded, by other pyogenic organisms such as staphylococci or streptococci. A post-typhoidal empyema of the gall bladder, for example, may be detected and relieved by incision and drainage. The purulent bile that comes away will contain the bacillus typhosus in pure culture. The sinus will go on discharging for weeks or months; cultivations made from the discharge from time to time will still give pure growths of the typhoid bacillus. Presently the surface opening will close, and the patient will seem to have been cured. She will go about her work, unavoidably exposed to chances of secondary infection; not infrequently there will be a re-accumulation of purulent bile, which will find its own way to the surface, and the patient will again come under observation with the discharging sinus covered by some homely bandage or a handkerchief; yet cultivations will often show that the bacillus typhosus is still uncontaminated.

In this connection it may be noted that urotropine is proved beyond doubt to be excreted both by the liver and by the walls of the gall bladder just as it is by the kidneys; it is a drug which inhibits the rate of multiplication of bacteria in the fluids and passages through which it is excreted; and therefore it is strongly indicated as a routine medicine to be given in all cases of infection of the gall bladder, and this whether the latter has been operated upon or not. The exhibition of urotropine in ten or fifteen grain doses thrice or four times daily is likely to assist very much in diminishing the infectiveness of the bile, and therefore in accelerating the rate of closure of the sinus of a gall bladder that has been opened and drained for empyema. It is also likely to diminish the risk of re-accumulation of infected bile, and thus minimise the chances of spontaneous re-opening of the sinus when it has once closed. The importance of this is clear when one has seen a case of post-typhoidal biliary fistula alternately opening and closing for months or years, possibly necessitating the use of a rubber-bag dressing for the reception of the bacillus-laden bile.

The way in which typhoidal suppuration may go on for years was well exemplified in a case of pyelitis recently described in this journal. There the patient had had pyuria, with typhoid bacilli in his urine, persistently for over ten years.

Another point about post-typhoidal suppuration is that it may not manifest itself for months after the patient has recovered from the fever. There was a case of sub-mammary abscess, for example, in which the patient was in a different country to that in which she had had her typhoid fever; she consulted a medical man, and said nothing about her illness of months before, attributing her breast trouble entirely to a recent blow. The abscess was opened and drained; there were doubts as to whether it might not be due to a tuberculous perichondritis, and cultivations were made from the pus. The bacterial growth that resulted consisted of typhoid bacilli in pure culture, and it was then that, taxed with having had enteric fever, the patient acknowledged at once that she had.

### The Disappearance of Widal's Reaction.

It might have been expected that Widal's blood-serum test would afford considerable evidence as to whether or not the patient had had typhoid fever previously; but it has been shown by M. G. Louisson, Herbert French and others that the blood loses its power of agglutinating typhoid bacilli very rapidly after the fever itself is past. In many cases the serum test has become negative again in less than three months from the time when it was strongly positive during the illness; in the great majority of persons who have had typhoid fever a year or more ago Widal's test is indeterminate or negative. Whether this is equally true in cases where there is post-typhoidal suppuration has not been fully investigated; but it is quite certain that, even if such suppuration may increase the chances of the serum reaction persisting partially or completely, in some instances the agglutinating power of the blood passes off, even though there are foci containing typhoid bacilli in the body. Agglutinative power of the blood seems to be quite a different thing to immunity against infection.

### Periosteal Nodes.

Periosteal nodes may occur during the actual fever, but it is important to realise that they may not develop for several weeks or months afterwards. In appearance and size they are not at all unlike those due to syphilis, and an error of diagnosis is very easily made when the nodes appear during or after convalescence. Their commonest situation is the front of the tibia, but no bone is exempt. The periosteum of the femur, the ribs, sternum, humerus, clivicle, and so on may be affected, and as a rule a patient who has one or more post-typhoidal nodes upon a tibia will also have one or more in connection with other bones about the same time. The affection is very painful in some cases, and it is usually associated with pyrexia. Each node is a potential abscess, but it is surprising how often the inflammation subsides without any definite pus being formed, and without any surgical measures being required. It may be followed by permanent local hypertrophy of the bone; but it is quite possible for even a big node to go away in a few weeks, leaving no residual effects.

When the node goes on to the formation of an abscess the pain becomes increased, swelling and redness of the affected part augment, until finally the knife has to be used. In view, however, of the possible resolution of the trouble, resort to the knife should be postponed as long as possible. The organisms recovered from the pus are usually typhoid bacilli in pure culture, though it occasionally happens that staphylococci or streptococci have been absorbed into the system, the node being then an evidence of secondary pyæmia. In a case recorded by Buschke the node and abscess did not develop until *forty-six years* after the typhoid fever, yet typhoid bacilli in pure culture were recovered from the pus.

